# Radiology appointment management in German hospitals: a survey of referring physicians

**DOI:** 10.1186/s13244-026-02303-7

**Published:** 2026-05-28

**Authors:** Philipp Reschke, Leon D. Gruenewald, Christian Booz, Ibrahim Yel, Simon S. Martin, Vitali Koch, Jennifer Gotta, Elena Höhne, Tatjana Gruber-Rouh, Katrin Eichler, Thomas J. Vogl, Andreas Michael Bucher

**Affiliations:** Department of Diagnostic and Interventional Radiology, Goethe University Hospital Frankfurt, Frankfurt am Main, Germany

**Keywords:** Radiology workflow, Appointment scheduling, Healthcare efficiency, Physician survey, Diagnostic imaging

## Abstract

**Objectives:**

Referring physicians’ experiences with imaging coordination remain underexplored. This study compares imaging coordination experiences between general practitioners (GPs) as outpatient referrers and hospital-based physicians as inpatient referrers.

**Materials and methods:**

A nationwide cross-sectional survey was conducted in Germany (June 2023–June 2024), including 220 physicians in the final analytical cohort (79 GPs, 141 hospital-based physicians).

**Results:**

GPs had significantly more professional experience than hospital-based physicians (20 ± 14 vs. 7 ± 9 years; *p* < 0.001), with no gender differences (*p* = 0.71). GPs rated the impact of imaging delays on patient care (4.4 ± 1.5) and coordination challenges with radiology services (4.6 ± 1.4) significantly above the neutral midpoint of 4.0 (whereas hospital-based physicians did so only for coordination challenges. Hospital-based physicians preferred real-time workflow tracking (34.8% vs. 9.0%; *p* < 0.001) and automated reminders of radiological appointments (20.6% vs. 6.5%; *p* = 0.006), whereas GPs favored centralized scheduling (33.3% vs. 19.2%; *p* = 0.02) and urgent case prioritization (42.3% vs. 23.4%; *p* = 0.005). Short-term appointment availability was the highest-ranked priority among the five evaluated categories by referring physicians, accounting for 25.0% of weighted rankings (*p* < 0.001).

**Conclusions:**

Scheduling delays remain a major barrier to timely diagnostics and reflect multifactorial system constraints. Referrer-specific appointment strategies may improve coordination across outpatient and inpatient settings.

**Critical relevance statement:**

This survey identifies barriers to radiology appointment scheduling among outpatient and inpatient referrers in German hospital radiology departments, providing a basis for targeted strategies to reduce imaging delays.

**Key Points:**

- Short-term availability ranks highest priority across groups of referrers.
- GPs reported higher perceived impact of imaging delays on patient care than hospital doctors.
- Referrer preferences vary: GPs favor centralized scheduling. Hospital-based physicians prefer real-time workflow tracking.
- Referrer-specific interventions may improve perceived reliability of imaging coordination.

**Graphical Abstract:**

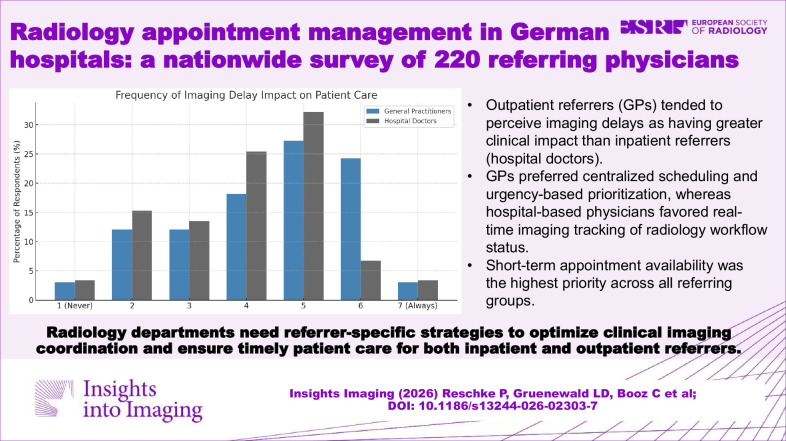

## Introduction

Radiology appointment scheduling involves coordinating imaging examinations to balance capacity, efficiency, and timely access to diagnostic services. As imaging demand continues to rise across Europe, radiology departments in hospitals face persistent constraints related to limited scanner availability and the need to reserve capacity for urgent examinations. Traditional centralized scheduling models often struggle to accommodate fluctuating workloads and dynamic clinical priorities [1–3]. Radiology departments have historically grappled with the challenge of balancing steadily increasing imaging demand against finite resources [4–6]. As healthcare systems evolve, departments must continuously adapt to accommodate growing case volumes, an increasing proportion of complex imaging studies, and the integration of advancing technological standards while maintaining efficient workflow management [7–9].

Across European healthcare systems, prolonged waiting times for imaging services, particularly magnetic resonance imaging (MRI), represent a persistent and pervasive challenge [10–12]. These delays are even more pronounced in low- and middle-income countries, creating significant global health inequities [13, 14]. Consequently, healthcare system efficiency is at stake. Across settings, delayed access to imaging can contribute to downstream scheduling pressure and prolong diagnostic pathways, particularly when coordination depends on multiple clinical and logistical interfaces. Longer waiting times may also reduce patient satisfaction and the perceived reliability of care delivery [15, 16].

The relationship between appointment delays and patient behavior creates additional operational challenges. Daye et al demonstrated that longer waiting times for imaging procedures were significantly associated with an increased likelihood of missed appointments [17], highlighting no-shows as a major challenge for radiology workflow management [18, 19]. As the primary interface between patients and imaging services, referring physicians play a pivotal role in facilitating appropriate imaging utilization [20].

Understanding the challenges and requirements of referring physicians in coordinating radiology appointments provides essential insights for enhancing scheduling efficiency. This study examines the perspectives and difficulties encountered by general practitioners (GPs) as outpatient referrers and hospital-based physicians as inpatient referrers in managing radiology appointments in German hospitals, thereby providing insights that may inform evidence-based strategies for workflow optimization.

## Methods

### Study design and setting

This cross-sectional survey study focused exclusively on physicians’ perspectives regarding appointment management in hospital radiology departments. GPs were included to capture outpatient referral processes involving German hospital radiology departments, whereas hospital-based physicians were included to reflect inpatient radiology workflows within these hospitals. The study was conducted in accordance with institutional ethical standards and local data protection regulations. No patient data were collected.

### Participants and recruitment

Between June 2023 and June 2024, we carried out a nationwide recruitment of actively practicing physicians who routinely referred patients for radiological imaging. To achieve a balanced sample, we used a stratified sampling strategy encompassing all 16 federal states of Germany, including both urban and rural areas. Hospital-based physicians were selected from high-referral specialties such as internal medicine, surgery, emergency medicine, and oncology. GPs were identified from registries maintained by regional medical associations and professional directories, using systematic sampling at defined intervals. In total, 1000 physician contacts were screened, and 453 eligible physicians were invited to participate based on predefined strata reflecting geographic and specialty distribution.

Of these, 280 physicians submitted the online survey. After applying the exclusion criteria 220 participants were included in the final analysis.

### Inclusion and exclusion criteria

Licensed physicians were actively included in the study. Exclusion criteria comprised retired physicians, individuals not involved in radiological referrals, and participants who provided only demographic information and failed to answer at least one main survey question.

### Survey development and validation

We developed a comprehensive survey instrument addressing appointment coordination challenges and improvement strategies. Prior to distribution, a pilot test was conducted with 10 physicians to evaluate clarity, item comprehensibility, and completion time. Feedback from this pilot phase was used to refine wording and optimize question flow. This process served as a pretesting phase.

The finalized survey was distributed electronically, with a reminder email sent 4 weeks later to optimize response rates. To minimize ordering bias, question blocks were randomized for each participant, while the demographic section remained fixed. All responses were collected anonymously.

### Survey domains

The survey evaluated several key domains. First, it collected demographic data, including professional experience, medical specialty, and field of work. Second, participants rated the perceived impact of imaging delays on patient care, defined as the extent to which postponed examinations affected diagnostic decision-making, treatment initiation, or overall clinical workflow.

Third, coordination difficulties were defined as challenges encountered during the process of arranging imaging appointments, including communication barriers, lack of available slots, and unclear scheduling responsibilities. Both constructs were assessed using 7-point Likert scales. Response anchors ranged from 1 = “never” to 7 = “always” for both impact on patient care and coordination difficulties. The midpoint (4.0) represented a neutral or moderate response. Additionally, physicians were asked to indicate their preferred strategies for improving coordination through a single-choice format. Finally, respondents prioritized various quality metrics relevant to radiology referrals (Supplementary Information). As not all participants answered every survey item, the number of responses can vary between questions.

### Real-time workflow tracking definition

**Fig. 1:**
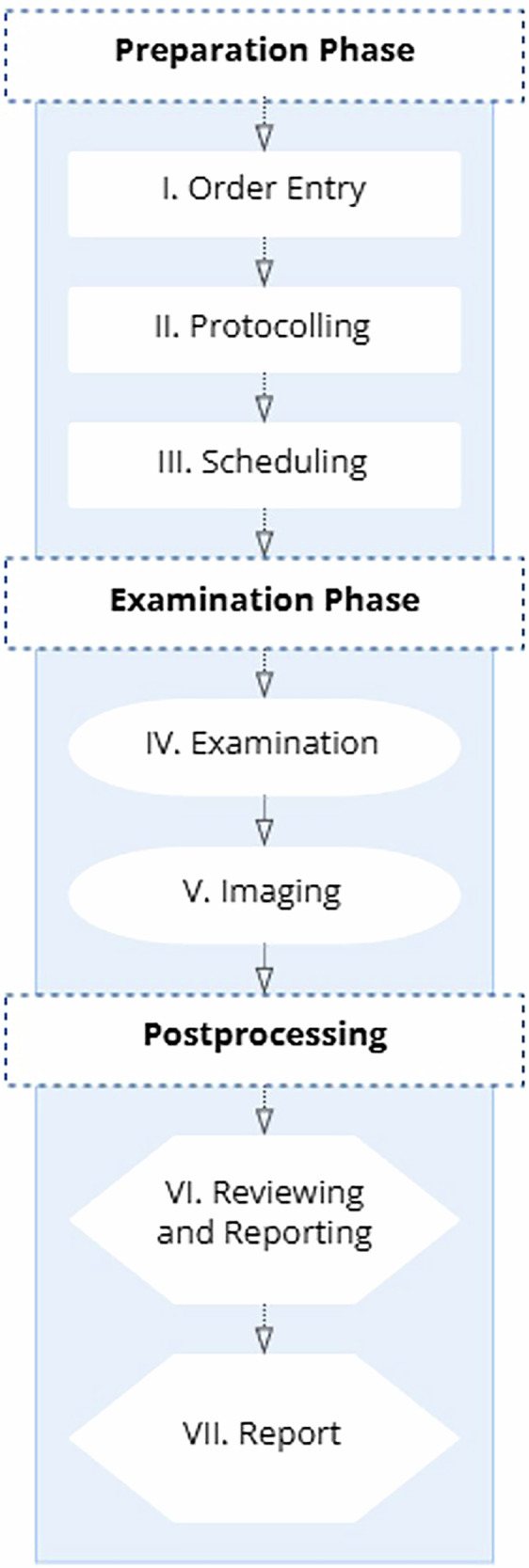
Workflow monitoring through real-time tracking in radiology. Conceptual framework illustrating the seven sequential stages of radiological workflow monitoring from order entry through report finalization. The diagram demonstrates how automated timestamp collection at each workflow stage (preparation phase: order entry, protocolling, scheduling; examination phase: patient examination, imaging acquisition; postprocessing: reviewing and reporting, final report approval) enables real-time identification of workflow bottlenecks and supports performance optimization initiatives.

In the survey, real-time tracking was presented as one potential strategy to improve radiology appointment coordination. It was briefly defined for participants as the continuous monitoring of core workflow steps — including referral submission, protocol assignment, scheduling, image acquisition, reporting, and report finalization. For conceptual clarification, Fig. 1 illustrates how real-time tracking may monitor key radiology workflow steps (Fig. 1).

### Statistical analysis

Continuous data are presented as means ± standard deviations. One-sample *t*-tests compared mean Likert scores against the neutral midpoint (4.0), while independent *t*-tests assessed between-group differences. Mann-Whitney U tests were additionally performed as non-parametric sensitivity analyses for between-group comparisons of Likert-scale variables. Chi-square tests evaluated categorical variable associations. For ranking analyses, total weighted scores per category were calculated by summing rank points assigned according to item position: in this five-item ranking question, first-ranked items received 5 points, second-ranked items 4 points, third-ranked items 3 points, fourth-ranked items 2 points, and fifth-ranked items 1 point. Chi-square goodness-of-fit tests determined whether specific priorities were significantly emphasized beyond chance expectation. Group differences of weighted scores between GPs and hospital-based physicians were evaluated using chi-square tests. Statistical significance was set at *p* < 0.05.

## Results

### Participant characteristics

**Fig. 2:**
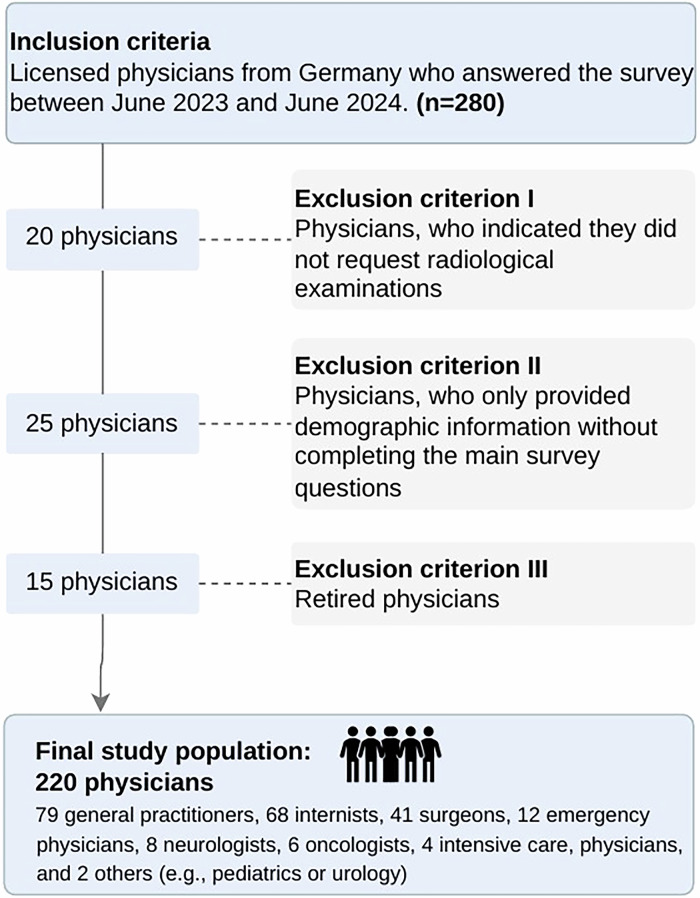
Inclusion and exclusion criteria. CONSORT-style participant flow diagram illustrating the recruitment and selection process for the study. From an initial pool of 453 eligible physicians invited to participate, 280 submitted the online survey (response rate: 61.8%). After applying predefined exclusion criteria—20 physicians who did not request radiological examinations, 25 with missing responses in the main survey part, and 15 retired physicians—a total of 220 participants were included in the final analysis. The analytical cohort comprised 79 GPs and 141 hospital-based physicians, including 68 internists, 41 surgeons, 12 emergency physicians, eight neurologists, six oncologists, four intensive care physicians, and two physicians from other specialties

Of the 453 physicians invited to participate, 280 submitted the online survey (response rate 61.8%). Exclusion criteria were applied to 280 physicians as shown in the flow chart. A total of 60 submissions were excluded: 20 physicians who did not order radiological examinations, 25 with incomplete responses, and 15 who were retired. The final analytical cohort, therefore, comprised 220 physicians: 79 GPs, who primarily refer outpatients to hospital-based radiology departments, and 141 hospital-based physicians, who coordinate imaging for inpatients within hospital settings. The hospital-based physician group included 68 internists, 41 surgeons (including trauma and orthopedics), 12 emergency physicians, eight neurologists, six oncologists, four intensive care physicians, and two from other specialties (Fig. 2). A total of 141 hospital-based physicians completed the survey, including 95 residents, 42 senior physicians, and 4 chief physicians. GPs demonstrated significantly greater professional experience than hospital-based physicians (20 ± 14 vs. 7 ± 9 years; *p* < 0.001). Gender distribution was comparable between groups, with males comprising 58.2% of hospital-based physicians and 60.8% of GPs (*p* = 0.71) (Table 1).

**Table 1:** Demographic characteristics of study participants

Group	Number of participants (n)	Male (n)	Female (n)	Years of experience	Standard deviation experience
Hospital-based doctors	141	82	59	7	9
GPs	79	48	31	20	14

Comparison of baseline demographic characteristics between GPs and hospital-based physicians. Years of experience represent total professional experience from completion of medical training. Statistical significance was assessed using independent *t*-tests for continuous variables and chi-square tests for categorical variables. *p* < 0.001 indicates statistically significant difference in years of experience between groups, while gender distribution showed no significant difference (*p* = 0.71)

### Impact of imaging delays on patient care

**Fig. 3:**
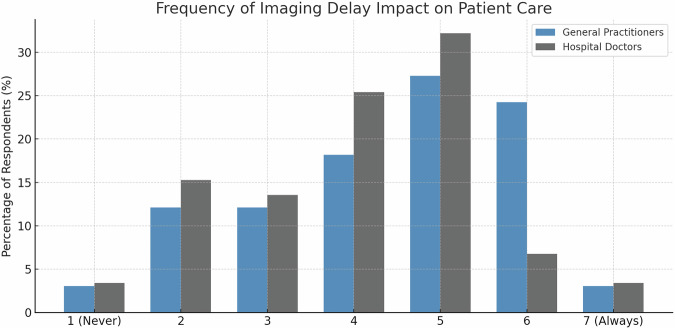
Perceived impact of imaging delays across physician groups. Frequency distribution of physician responses regarding the impact of imaging delays on patient care, measured on a 7-point Likert scale (1 = never impacts care, 7 = always impacts care). GPs (*n* = 73) are represented by blue bars and hospital-based physicians by gray bars. Statistical comparisons were performed using one-sample *t*-tests against the neutral midpoint (4.0). GPs rated the impact of imaging delays on patient care significantly above the neutral midpoint, while hospital-based physicians (*n* = 141) showed no significant deviation from neutrality

GPs reported that imaging delays impact patient care more frequently than hospital-based physicians (mean = 4.4 ± 1.5 vs. 4.0 ± 1.5 on a 7-point scale). Compared to the neutral midpoint (4.0), only GPs rated the impact as significantly above the neutral point (one-sample *t*-test, *p* = 0.03), while hospital-based physicians showed no significant deviation from the neutral point (one-sample *t*-test, *p* = 0.8) (Fig. 3).

### Coordination challenges

**Fig. 4:**
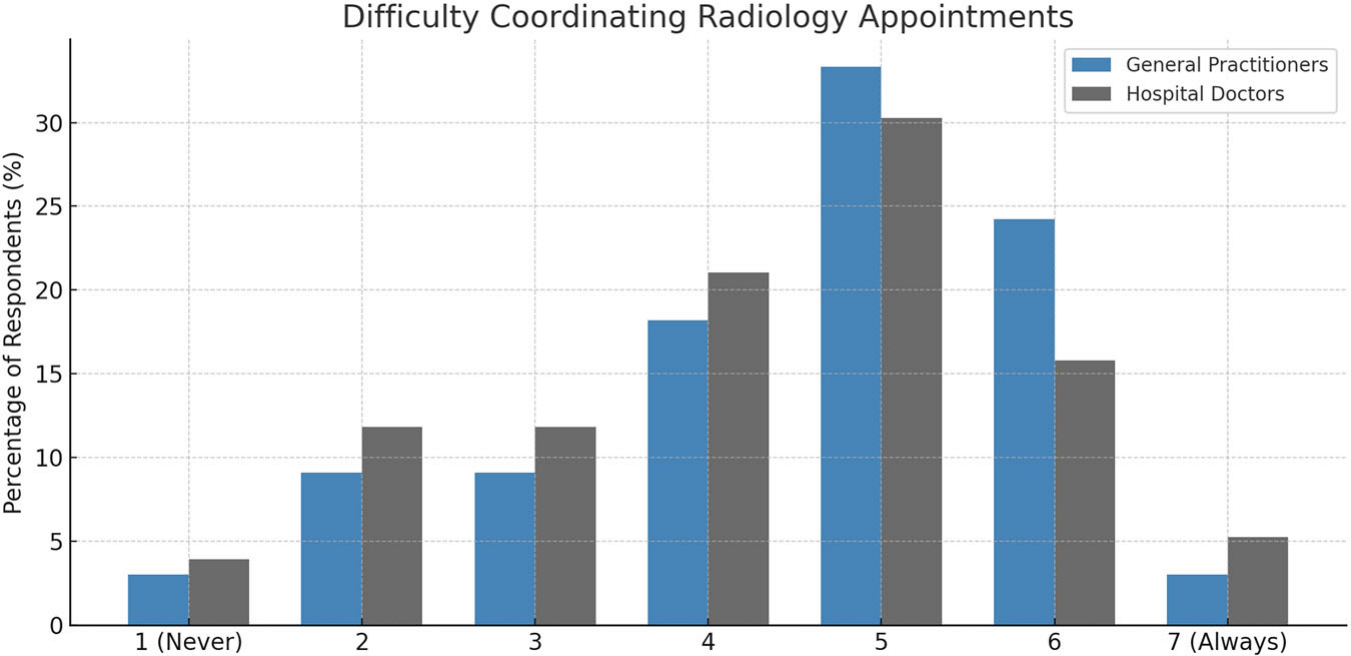
Challenges in scheduling radiology appointments. Distribution of coordination difficulty ratings on a 7-point Likert scale (1 = no difficulties, 7 = severe difficulties) comparing GPs (blue bars, *n* = 77) and hospital-based physicians (gray bars, *n* = 141). Data analysis included one-sample *t*-tests against the neutral midpoint of 4.0 and independent *t*-tests for between-group comparisons.

GPs experienced slightly higher coordination difficulties with radiology services compared to hospital-based physicians (mean = 4.6 ± 1.4 vs. 4.3 ± 1.5), although the between-group difference was not statistically significant (independent-samples *t*-test, *p* = 0.209). Non-parametric sensitivity analyses using Mann–Whitney U tests yielded results consistent with the independent-samples *t*-tests. Compared with the neutral midpoint of 4.0, ratings were significantly higher in both groups, with a stronger deviation among GPs (GPs: one-sample *t*-test, *p* < 0.001; hospital-based physicians: *p* = 0.02) (Fig. 4).

### Preferred improvement strategies

**Fig. 5:**
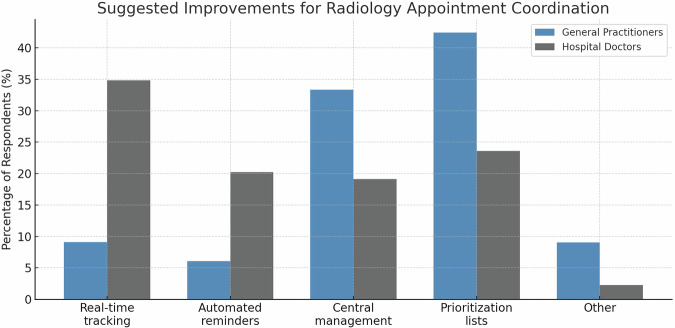
Referrer-specific approaches to optimize appointment scheduling. Comparative analysis of preferred improvement strategies for radiology appointment management, presented as percentage of respondents selecting each intervention. GPs (*n* = 78) are represented by blue bars and hospital-based physicians (*n* = 141) by gray bars. Statistical significance was assessed using chi-square tests for categorical variables. Hospital-based physicians demonstrated significantly greater preference for technology-based solutions (real-time tracking and automated reminders), while GPs favored organizational interventions (centralized management and prioritization protocols)

Significant differences emerged in improvement preferences between referring physician groups. Hospital-based physicians demonstrated significantly greater preference for real-time workflow tracking (34.8% vs. 9.0%; chi-square test, *p* < 0.001) and automated appointment reminders for patients and referring physicians (20.6% vs. 6.5%; *p* = 0.006). Conversely, GPs significantly favored centralized appointment management systems (33.3% vs. 19.2%; *p* = 0.02) and prioritization protocols for urgent examinations (42.3% vs. 23.4%; *p* = 0.005) (Fig. 5).

### Quality metrics prioritization

**Fig. 6:**
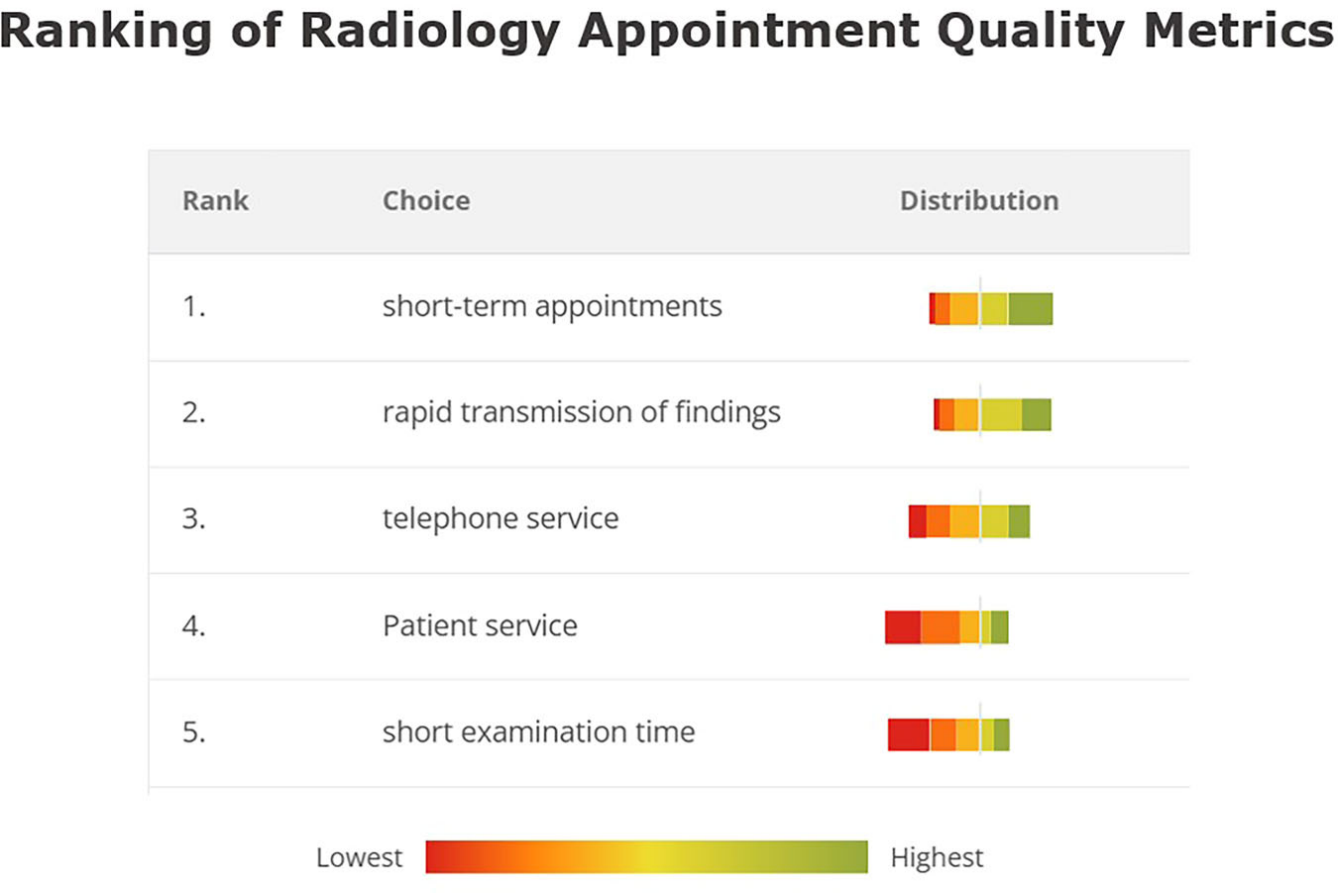
Top-ranked factors for effective radiology scheduling. Weighted ranking analysis of quality metrics deemed most important for effective radiology appointment management. Results are presented as percentage of total weighted scores, with color gradients indicating preference intensity from lowest (red) to highest (green) priority. Analysis included chi-square goodness-of-fit tests to assess whether priorities exceeded chance expectation. Short-term appointment availability emerged as the universally highest-ranked priority for GPs (*n* = 79) and hospital-based physicians (*n* =141).

Across all respondents, the distribution of weighted ranking scores differed significantly from an equal distribution across the five categories (*p* < 0.001), with short-term appointment availability receiving the highest share of total weighted scores (25.0%), followed by rapid report transmission (23.8%), telephone accessibility (20.4%), patient service quality (15.4%), and short examination time (15.4%). Category-wise comparisons showed that GPs assigned significantly greater relative weight to telephone accessibility than hospital-based physicians (22.5% vs. 19.2%; *p* = 0.025), whereas hospital-based physicians assigned significantly greater weight to short examination time than GPs (16.6% vs. 13.1%; *p* = 0.006). No significant group differences were observed for short-term appointment availability between GPs and hospital-based physicians (26.0% vs. 24.4%; *p* = 0.310), rapid report transmission (23.5% vs. 24.0%; *p* = 0.718), or patient service quality (14.9% vs. 15.7%; *p* = 0.562) (Fig. 6).

## Discussion

This study provides comprehensive insights into referring physicians’ perspectives on radiology appointment management. To our knowledge, the perspectives of referring physicians on radiology appointment management have received limited attention despite their central role in ensuring workflow efficiency.

Our analysis revealed three principal findings. First, on the 7-point scales, referring physicians reported moderate levels of coordination challenges (GPs: 4.6 ± 1.; hospital-based physicians: 4.3 ± 1.5) and perceived impact of imaging delays on patient care (GPs: 4.4 ± 1.5; hospital-based physicians: 4.0 ± 1.5). Second, improvement preferences differed significantly between groups: hospital-based physicians favored real-time work flow tracking (34.8% vs. 90%, *p* < 0.001) and automated reminders (20.6% vs. 6.5%, *p* = 0.006), while GPs preferred centralized management (33.3% vs. 19.2%, *p* = 0.02) and prioritization systems (42.3% vs. 23.4%, *p* = 0.005). Third, short-term appointment availability was universally prioritized as the most important scheduling metric, within an overall distribution that differed significantly across scheduling metrics (*p* < 0.001).

The reported coordination challenges—particularly among GPs—may reflect system-level communication constraints, including limited availability of structured points of contact, variable workflow interfaces, and competing clinical priorities. These barriers likely contribute to GPs’ strong preference for centralized appointment management, which may provide clearer access routes and standardized communication pathways. While structural improvements are essential, these challenges likely reflect shared systemic pressures across disciplines rather than individual shortcomings, underscoring the importance of collaborative approaches to radiology workflow management. However, such measures require addressing escalating costs for personnel, while expenses for advanced equipment like high-end CT scanners far outpace stagnant reimbursement rates for examinations [21–23]. To address this, the implementation of structured call triage systems—utilizing artificial intelligence or trained personnel—could bridge this communication gap efficiently [24–28].

The higher perceived impact of imaging delays among GPs likely reflects longer waiting times in outpatient compared with inpatient settings. Previous research at University Hospital Mannheim demonstrated that outpatients experienced 44-day average waiting times for MRI compared to 3 days for inpatients [12].

This disparity underscores the need for equitable access strategies that address the unique challenges faced by GPs. Payment structures and reimbursement models may represent relevant factors influencing resource allocation between inpatient and outpatient settings. In this context, inpatient imaging is not reimbursed directly but is funded indirectly through Diagnosis-Related Group (DRG) payments to the hospital, with budgets for radiology departments determined internally as part of the hospital’s resource allocation process [29]. These economic dynamics might incentivize hospitals to prioritize inpatient imaging workflows to support timely discharge and optimize DRG-based revenue [30], but this relationship was not directly assessed in the present study. More broadly, scheduling delays should be interpreted within a multifactorial system context, including administrative burden, workforce limitations (e.g., radiologists, technologists, transport and nursing staff), and variation in exam appropriateness and prioritization practices. As this survey captures referrer perceptions rather than objective capacity or appropriateness metrics, these interpretations should be considered hypothesis-generating. Complementary approaches—such as clearer referral criteria, structured mechanisms to address potentially inappropriate requests, and protocol optimization to maintain diagnostic quality while improving throughput—may help mitigate delays but require confirmation in studies incorporating objective workflow data. Given differences in reimbursement and care pathways across European health systems, the operational principles identified here (e.g., transparent prioritization, defined communication routes, and referrer-tailored scheduling tools) should be interpreted cautiously and adapted to local contexts.

GPs’ preference for prioritization systems may reflect difficulties navigating fragmented hospital workflows. Transparent referral and prioritization processes may complement direct GP access. Previous evidence suggests that GP-focused direct access initiatives can improve healthcare delivery, patient care, waiting times, and referral processes [31]. Implementing structured intake pathways—supported by trained administrative staff, standardized decision rules, and digital tools where available—might reduce interruptions and clarify escalation routes for time-critical cases. However, feasibility depends on adequate staffing, infrastructure, and alignment with local reimbursement and capacity constraints. In one radiology call-setting study, a call triage assistant handled most telephone calls without disturbing the on-call resident, reducing interruptions by 71%, shortening report turnaround times, and lowering resident-reported stress and distraction [27].

Hospital-based physicians’ preference for real-time tracking reflects the dynamic nature of inpatient care. Evidence supports that real-time tracking systems can reduce report delays by up to 38% [32]. A persistent challenge observed in clinical practice involves reports being routed to radiologists who are unavailable for final approval, resulting in workflow bottlenecks and delayed report turnaround [9]. Intelligent routing systems could automatically track radiologist availability and direct reports to present staff members, thereby minimizing delays. Although real-time tracking and intelligent routing systems require substantial upfront investment, they may yield long-term economic benefits through improved throughput, reduced report turnaround times, and lower no-show rates, ultimately offsetting implementation costs [32, 33].

The emphasis on automated appointment reminders by hospital-based physicians addresses the significant challenge of missed appointments. No-show rates reach 19.3% in European facilities, 23.5% in North American centers, and 43% in South American institutions [34, 35]. Evidence consistently demonstrates that reminder systems effectively reduce no-show rates across healthcare settings [36–38]. Outpatient imaging appointments in hospital-based departments—such as oncology or neurology—must be coordinated alongside complex inpatient workflows, and automated reminders for patients and referring physicians might support clinicians in maintaining oversight of parallel care pathways and reducing scheduling disruptions.

The universal prioritization of short-term appointment availability indicates a shared commitment to reducing diagnostic delays across all referring physician groups. This can be operationalized through dedicated time slots for high-volume, standardized examinations. Body region-specific scheduling—such as dedicated knee MRI slots—enables workflow optimization through standardized protocols and specialized staff training, enhancing both efficiency and image quality [39, 40].

To sustain these improvements, radiology departments may benefit from structured quality management approaches such as Kaizen—a Japanese philosophy emphasizing continuous, incremental improvement involving all stakeholders [41]. For example, radiology departments facing MRI delays could hold regular Kaizen workshops to identify inefficiencies and implement targeted improvements such as optimized triage or time slot allocation.

This study has several important limitations. First, the use of structured survey questions limited deeper qualitative insights that might emerge from open-ended interviews. Second, the study relied solely on self-reported perceptions without integrating objective workflow metrics or scheduling data. Third, although Likert-scale data are ordinal, parametric tests were used given the large sample size, assuming interval scale properties. Fourth, the cross-sectional design prevents assessment of temporal changes in perceptions or system performance. Fifth, the survey did not capture aspects such as prognostic shortfall or low-value imaging. These dimensions cannot be reliably assessed through self-reported perceptions, as identifying low-value examinations requires objective linkage between imaging indications, findings, and subsequent patient outcomes. Sixth, although the overall response rate of 61.8% is relatively high for physician surveys, potential non-response bias cannot be excluded. It is possible that physicians with stronger opinions or greater frustration regarding radiology scheduling were more motivated to participate, while those with fewer coordination challenges may have been underrepresented. Seventh, while real-time tracking was frequently cited as a preferred improvement strategy, its implementation remains challenging, as it requires substantial IT infrastructure, interdepartmental coordination, and data standardization across scheduling, imaging, reporting, and communication processes. Eighth, given the exploratory nature of this survey and the assessment of multiple related outcomes, *p*-values should be interpreted cautiously. Accordingly, the reported inferential statistics are intended to identify patterns rather than to provide confirmatory evidence. Finally, this survey reflects referrer experiences within the German healthcare system and does not capture broader European factors such as administrative constraints, workforce variability, or differences in imaging utilization and system priorities. Future research should evaluate tailored interventions that improve efficiency and care coordination using objective workflow metrics alongside physician-reported experiences. In particular, implementation studies assessing the feasibility, effectiveness, and cost-effectiveness of scheduling optimization strategies will be important to guide sustainable, evidence-based adoption.

In summary, our findings highlight the need for referrer-specific strategies in radiology appointment management. Delays and coordination challenges persist across all groups, with short-term appointment availability as a shared priority. Real-time tracking, centralized scheduling, and prioritization protocols may help address these needs.

## Supplementary information

ELECTRONIC SUPPLEMENTARY MATERIAL

## Data Availability

Data supporting the findings of this study are included within the manuscript, and the raw data are available from the corresponding author upon reasonable request.
