# Supplementary material for: Radiology appointment management in German hospitals: a survey of referring physicians
Source: Insights Imaging. 2026 May 28;17:144. doi: 10.1186/s13244-026-02303-7 (PMC13219557; doi:10.1186/s13244-026-02303-7)
Supplement: Supplementary file 1 — ELECTRONIC SUPPLEMENTARY MATERIAL [file 13244_2026_2303_MOESM1_ESM.pdf]

Radiology Appointment Management in German Hospitals: A Survey of Referring Physicians

ELECTRONIC SUPPLEMENTARY MATERIAL

Referring Physicians' Perspectives on Radiology Appointment Management

What field do you work in?

Please choose...

How many years of professional experience do you have?

#

0 / 80

Please select your field of activity.

- Practice doctor, who regularly refer outpatients to hospital-based radiology departments
- Resident physician in a clinic, who coordinate imaging for inpatients
- Senior physician in a clinic, who coordinate imaging for inpatients
- Chief physician in a clinic, who coordinate imaging for inpatients
- None of this is true

How often do delays in hospital imaging affect diagnostic decisions, treatment initiation, or the overall management of your patients?

Never | Always

1	2	3	4	5	6	7

How often do you have difficulties arranging radiological examinations for your patients in a hospital radiology department – for example, due to poor telephone accessibility, limited appointment capacity, or unclear responsibilities?

Never | Always

1	2	3	4	5	6	7

In your opinion, how could appointment coordination in radiology in hospitals be improved?

(Please select the measures you consider appropriate. Brief explanations of each option are provided in parentheses.)

- ○ Real-time tracking

  (Real-time tracking in radiology refers to the continuous monitoring of core workflow steps—including referral submission, protocol assignment, scheduling, image acquisition, reporting, and report finalization—using automated time stamps to detect delays and optimize throughput.)

- ○ Automated appointment reminders for patients and physicians

  (Patients and referring physicians can decide whether they want to use the reminder service and in what form they would like to receive the reminder, SMS or email.)

- ○ Central appointment management

  (Central appointment management implemented in the form of a specialized team that coordinates appointment scheduling and inquiries in a uniform manner.)

- ○ Creation of prioritization lists

  (It is a digital display that transparently shows a patient's current prioritization status. This ensures an efficient and transparent sequence of examinations.)

- ○ other

Please rank the following aspects of radiological appointment scheduling in hospitals according to your personal importance – starting with 1 (most important) to 5 (least important):

short-term appointments
(e.g. as soon as possible after transfer)

1.

telephone availability
(e.g. for queries or appointment changes)

Patient service
(e.g. information session before the examination, discussion of findings)

short examination time
(e.g., the imaging process itself should be as fast as possible)

rapid transmission of findings
(e.g. prompt delivery of the report to you or the patient)
